# 

**Published:** 1921-08-20

**Authors:** 


### Presentation to Asylum Superintendent.

Dr. T. E. Knowles Stansfield, C.B.E., the first medical superintendent of Bexley Mental Hospital, has resigned the position after twenty-three years' service. On the occasion of his formal farewell, which was attended by a large number of visitors, a handsome silver-gilt casket was presented to him from the officers and staff of the institution containing an address. Dr. C. Hubert Bond, one of the Commissioners in Lunacy and first senior medical assistant at Bexley, attended the presentation.

### The Miller Hospital.

The total received or promised for the extension fund of the Miller General Hospital, part of which will be a war memorial, is £45,000. The need for new nurses' quarters is urgent, for which £20,000 is required. The hospital now boasts fourteen special departments, is increasing its income from employees' contributions, and steadily extending its organisation of collections in schools. Payments from out-patients at the rate of one shilling for each attendance have also been suggested.

### Forthcoming Publications.

MESSRS. WILLIAM HEINEMANN (Medical Books), Ltd., have in preparation and will shortly publish the following books: "Special Pathology" (second edition), by J. Martin Beattie, M.D., Professor of Bacteriology, University of Liverpool, and W. E. Carnegie Dickson, M.D., Director of the Pathological Department Royal Hospital for Chest Diseases, London; "The Surgical Exposure of the Deep-seated Blood-vessels," by J. Fiolle, M.D., and J. Delmas, M.D., translated and edited by Charles Greene Cumston, B.S.M., M.D., with a Preface by Sir D'Arcy Power, K.B.E.; "The Surgical Treatment of Non-Malignant Affections of the Stomach," by Charles Greene Cumston, M.D., and George Patry, M.D., Lecturers at the University of Geneva, and members of the Surgical Society of Switzerland, with an Introduction by Sir Berkeley G. A. Moynihan, K.C.M.G.

MESSRS. A. & C. BLACK announce a third edition, revised and brought up to date, of a "Text-book of Midwifery," by R. W. Johnstone, C.B.E., M.D., F.R.C.S.E., containing 262 illustrations in the text. The short period within which these three editions have been called for is testimony to the popularity of the book.

### Medical Appointments.

MR. KENNETH BELLWOOD, O.B.E., F.R.C.S., has been appointed Surgeon to the Albert Dock Hospital of the Seamen's Hospital Society.

DR. S. RIDDIOUGH, of Earlsheaton, has been appointed to the staff of the Dewsbury and District General Infirmary.

DR. HARRY MIDDLETON, deputy county medical officer and chief assistant medical officer, Denbighshire, has been appointed county medical officer of health and school medical officer for the county of Pembroke.

MISS MARY O'CONNOR, M.B., B.Ch., has been appointed assistant school medical officer to the County Borough of Plymouth. Miss O'Connor is a graduate of University College, Cork.

### Help in Appeal Work.

THE generous offer of the Roneo Co., Ltd., to supply one of their duplicators free to hospitals in all parts of Great Britain and in a number of Continental countries, where good use can be made of the machine, has been accepted to the extent of about 300 machines. At present this must be the limit of the company's offer, and no further requests can be fulfilled, though the directors inform us they are quite willing for hospitals to communicate with them on the subject on the understanding that their requests will be granted as soon as the company can conveniently do so.

### Ministry of Health Annual Report.

As we go to press the annual report of the Ministry of Health for 1920-21 is issued by the Stationery Office (Cmd. 1,446, price 2s.). It covers the whole range of the activities of the Department, and is contained in a single volume. This step has been taken, it is said, "with the double purpose of reducing the cost of production and of enlarging the circle of those who may be prepared to buy and read a yearly account of some of those operations of Government which affect most closely their daily lives." We propose to deal with this report in a subsequent issue.

### The Liverpool Council of Voluntary Aid.

THE Quarterly Paper (July) of the Liverpool Council of Voluntary Aid is again an interesting one. It records a useful meeting, which was held in June, of treasurers of Liverpool charities to consider the increase in the assessment of charitable buildings. The charities will be severely hit by the additional sums required of them. The Assessment Committees are to be approached, and a deputation was appointed to meet the Joint Assessment Committee should that plan prove more acceptable. The Quarterly Paper also mentions that the Liverpool Council of Voluntary Aid's suggestions upon a uniform method of accounts for charities have been influential, and that "some of the Government Departments, in their recent regulations, have added requirements" embodying some of them. The Paper also draws attention to the World Service Exhibition which is to be held in St. George's Hall, Liverpool, from October 3-16. The five sections of the exhibition, which in the past two years has been held at Cambridge, Oxford, Huddersfield, Leeds, and Reading, show the need for service in the spheres of religion, preventive work (health), education, industry, and welfare. It calls for service and points the way. Any profits are given to local charities. At Reading £700 was distributed. New legislation, Government reports, and local special publications of interest to institutional officers are noticed.

### The Wellcome Historical Medical Museum.

THIS museum will be closed for cleaning and decorating from September 1 to September 30 inclusive.

### Matrons' and Sisters' Appointments.

Preston Royal Infirmary.—Miss Ellen Catherine Davis has been appointed sister. She was trained at the Leeds General Infirmary and the City Hospital, Nottingham, has been staff nurse and holiday sister at the Hospital for Paralysis and Epilepsy, Maida Vale, and temporary night sister and sister of the children's wards at the Hospital of St. Cross, Rugby. She is a member of the College of Nursing. Miss Hilda Jackson has been appointed sister. She was trained at the Preston Royal Infirmary, the Leeds City Hospital, Seacroft, and the Liverpool Maternity Hospital. She has taken sister's holiday duties at the first-mentioned hospital.

Farnham Union Infirmary.—Miss Bertha Brindley has been appointed ward sister. She was trained at Oldham Infirmary, where she was later sister; she has been senior staff nurse and staff midwife-in-charge at Ilkeston Maternity Hospital.

Royal Cornwall Infirmary, Truro.—Miss K. Blatchford has been appointed night sister. She was trained at the Royal Albert Hospital, Devonport, and has been staff nurse and later sister at the Coventry and Warwickshire Hospital.

Stockport Infirmary.—Miss L. M. Woolgar has been appointed night sister. She was trained at Charing Cross Hospital, and has been sister at the Craiglockhart War Hospital, Edinburgh, at the Craigronach Auxiliary Hospital, Troon, and at the Stockport Infirmary. She is a member of the College of Nursing.

Kelso Infirmary and Cottage Hospital.—Miss N. Deane Robertson, A.R.R.C., has been appointed matron. She was trained at Stobhill Hospital, Glasgow, and has since been nurse at Nordrach-on-Dee Sanatorium, Banchory, charge nurse at the District Hospital, Johnstone, and on the staff of the Aberdeen Nurses' Co-operation. She served four and a-half years in Q.A.I.M.N.S.R. as ward, theatre, and home sister and night superintendent, and as charge sister in the Ministry of Pensions Nursing Service. She is a member of the College of Nursing.

Stockport Maternity Home.—Miss D. M. Downes and Miss E. Paterson Machie have been appointed sister-midwives. Miss Downes was trained at the Birmingham Poor-Law Infirmary, and has since been health visitor at Plymouth and temporary night sister at Basford Infirmary. Miss Machie was trained at the West London Hospital and the Lord Mayor Treloar Hospital for Children. Between 1916 and 1920 she was on military service at home and abroad. Both hold the certificate of the Central Midwives Board.

Gloucester Royal Infirmary and Cheltenham Hospital.—Miss E. Cockayne has been appointed sister-tutor to these two hospitals. She was trained at Sheffield Royal Infirmary, where she was later ward and night sister. She has taken fever training at Plymouth Borough Hospital and midwifery training at Birmingham Maternity Hospital. Miss Cockayne is a member of the College of Nursing.

Westmorland Sanatorium, Grange-over-Sands. — Miss H. G. Hamilton has been appointed sister. She was trained at Blackburn Poor-law Institution, and has since been sister at Middleton-in-Wharfedale Sanatorium, and of the tuberculosis block at Nottingham General Hospital, also at Yardley Road Sanatorium, Birmingham.

North Devon Infirmary, Barnstaple.—Miss Lingard has been appointed night sister. She was trained at Northampton General Hospital, where she gained the certificate of the Chartered Society of Massage. She has been night sister at the Royal Hamadryad Seamen's Hospital, Cardiff, and temporary ward sister at Warrington Infirmary.

Albert Dock Hospital, E.—Miss L. C. Woolveridge has been appointed sister. She was trained at Great Yarmouth General Hospital, and has served in the Territorial Force Nursing Service.

Norwich Isolation Hospital.—Miss R. Menzies has been appointed home sister. She was trained at the Royal Alexandra Hospital, Paisley, and has since been staff nurse and sister at Norwich Isolation Hospital and matron of Turiff Red Cross Hospital. Miss Menzies is a member of the College of Nursing. Miss Florence Felt has been appointed night sister. She was trained at the Worcester General Infirmary and at the City Hospital, Little Bromwich, and has been ward sister at the Isolation Hospital, Norwich, and night sister at the General Hospital, Weston-super-Mare. She is a member of the College of Nursing.

Essex County Hospital, Colchester.—Miss Jean Brown has been appointed assistant matron. She was trained at the Essex County Hospital, where she has held the posts

(*Continued on p.* 358.)

### A Novel Gift and Others.

Recently the King Edward VII. Hospital and the Prince of Wales' Hospital for the Limbless at Cardiff have acknowledged substantial cheques which have come to hand under more or less unusual circumstances. At least one of the incidents may suggest opportunities to other well-disposed folk or to hospital secretaries on the look-out for new ideas for subscriptions. Mr. Leonard D. Rea, superintendent of the first-named institution, has received a cheque for 100 guineas from Messrs. James Howell & Co., a local firm, to commemorate the first annual meeting since the concern was formed into a public limited company. The same gentleman also acknowledges a cheque from Sir J. Herbert Cory, M.P., being a portion of his parliamentary salary, which he divides each quarter between the King Edward VII. Hospital and the Royal Hamadryad Hospital at Cardiff. In regard to the Prince of Wales' Hospital, a gentleman who had been absent from Cardiff for some time was glad to find an institution doing such good work for the crippled, and deposited his cheque for £25, on condition that his identity should not be revealed.

### Royal Waterloo Hospital.

A Sports Meeting and Gala, which it is hoped will raise sufficient money to enable building operations in the Royal Waterloo Hospital extension scheme to be resumed, is being organised by the Civil Servants, ex-Service officers and men, of Cornwall House, S.E. The Gala will take place at Durham Farm, Grove Park, S.E., on Saturday afternoon, September 3. As has been already announced, the building scheme has been temporarily abandoned owing to lack of funds, though £21,000 of the £80,000 appealed for has been received. It is hoped that gifts in kind, which it is proposed to sell by auction at the Gala, as well as donations, may be sent to the hospital.

### The Labour Hospital, Golder's Green.

The institution known as the Manor House Hospital, Golder's Green, which is managed by the Industrial Orthopædic Society, has held a Fête, at which the point of view of labour hospitals was expressed. Mr. J. Mills, M.P., said that, because the voluntary system was "bound to fail" sooner or later, they were anxious to form a ring of labour hospitals in all industrial districts, so that those who met with accidents or illness in the course of their work might be attended in hospitals run by and for Labour. Mr. C. J. Ammon said that a visit to the Manor House Hospital would show the ability of Labour to organise if it only received a fair chance to prove what it could do.

### The Guildford Saturday Workers.

The Mayor of Guildford, Alderman G. W. Franks, has entertained about sixty workers for the Guildford Hospital Saturday Fund, for which £630 has been collected. The villages raised £360 and the town £270. The work of the collectors was rewarded by the following honours: Mr. Cosh was made a Life Governor; Mr. W. H. Crawshaw a Representative Life Governor of the Surrey Convalescent Home, and Mrs. A. Stovold, of Puttenham, of the Victoria Convalescent Home for Women. Mrs. Stovold made the whole collection of Puttenham herself. Sir Edward Ellis, Treasurer of Guildford Hospital, in accepting a cheque for £500, said that such cheques were the best tonics which a Treasurer could have. These small pieces of paper represented an immense amount of trouble and personal service.

### Matrons' and Sisters' Appointments.

(*Continued from p.* 356.)

of staff nurse, sister, and theatre sister. Miss Mabel Hall has been appointed theatre sister. She was trained at Essex County Hospital, and has since held posts of district staff midwife at the General Lying-in Hospital, Lambeth, and sister of a women's surgical ward at her training school. She holds the certificate of the Central Midwives Board and of the National Hospital for massage and medical electricity.

Liberton Cottage Hospital.—Miss E. J. Thomson has been appointed night sister. She was trained at the Royal Alexandra Infirmary, Paisley, was sister in the Q.A.I.M.N.S.R., and has been sister at the Queen's Hospital, Sidcup, and at the Darrington Hospital, Pontefract.

Rochford Union.—Miss Edith Florence Jones has been appointed superintendent nurse. She was trained at Southampton Incorporation Infirmary, and has been superintendent nurse at the Infirmary, Rothwell Haigh, Leeds.

Maternity Hospital, Crewe.—Miss Florence Fox has been appointed matron. She was trained at Essex County Hospital, where she has held posts as sister, returning as assistant matron after an interval as maternity night sister at Dundee Royal Infirmary.

New End Hospital, Hampstead.—Miss Esther Fisher has been appointed matron. She was trained at Northampton General Hospital, later being ward sister there. She has been night sister at Lincoln County Hospital, night superintendent and class sister at Fulham Infirmary, night superintendent at Whipps Cross Hospital, and assistant matron of Mill Road Infirmary, Liverpool.

Northern Hospital, Manchester.—Miss Gertrude Parkinson has been appointed sister of the children's medical wards and Miss Constance Andrews theatre sister. Miss Parkinson was trained at the Leeds General Infirmary, and has been sister at the Eston Hospital, Middlesbrough, and at Tunbridge Wells Hospital. Miss Andrews was trained at the Royal Victoria and West Hants Hospital, Boscombe, and has been staff nurse at the Samaritan Hospital for Women, London, and ward sister and theatre sister at the Stroud General Hospital.

General Hospital, Altrincham.—Miss Sybil Bamlett has been appointed night sister. She was trained at the Royal Victoria Infirmary, Newcastle-on-Tyne.

Dudley Union Infirmary.—Miss Ann Jones has been appointed sister. She was trained at Shirley Warren Infirmary.

Hackney Infirmary.—Miss Alice M. Shotter has been appointed assistant matron. She was trained at Portsmouth Infirmary, subsequently taking her midwifery training. She has done holiday duty at Paddington Infirmary, was night sister at Dudley Infirmary, ward and night sister at Paddington Infirmary, massage sister at the Norfolk War Hospital, and assistant matron at the Booth Hall Infirmary, Manchester.

Johannesburg Hospital, South Africa.—Miss M. E. G. Milne has been appointed sister-tutor and sails early in September on a three years' agreement. The selection of candidates was made by the College of Nursing on the request of the Johannesburg Hospital. Miss Milne was trained at St. Thomas's Hospital and received the gold medal. Later she was awarded the Nightingale Scholarship at King's College for Women, where she took the sister-tutor's course, and subsequently held the post of sister-tutor at Queen Mary's Hospital, Stratford.

Isolation Hospital, Reigate.—Miss R. Ives has been appointed matron. She was trained at Portsmouth Union Infirmary, and has been matron of the Isolation Hospital at Dover and at Luton.

Raywell Sanatorium, Cottingham, Hull.—Miss Avis Lambert has been appointed sister. She was trained at the Chelsea Infirmary, where she was later sister. During the war she was attached to the Q.A.I.M.N.S.

### The College of Nursing.

**(Limited by Guarantee.)**

### SHEFFIELD CENTRE.

(Hon. Secretary, Mrs. Barnes, 34 Wilkinson Street.)

Death of a Member.

The death took place on August 2, at the Middlesex Hospital, after a painful illness, of Sister Ethel May Watson. Sister Watson served throughout the war on the staff of the 3rd Northern General Hospital (Territorial), where she was very highly respected. Her death will cause deep sorrow to all who knew her. A vote of condolence on behalf of the members of the Centre has been sent to her family.

### EDINBURGH CENTRE.

(Hon. Sec., Miss Cathcart, The Elms, Whitehouse Loan.)

A Sale of Work in aid of the Endowment Fund of the College of Nursing is to be held on Thursday, December 1, 1921. The following have consented to receive contributions in money or kind for the various stalls: *City Hospital*, Miss Thomas. *College Members*, Miss Cumming, Longmore Hospital; Miss Turnbull and Miss Cathcart, The Elms, Whitehouse Loan. *Mental Hospitals*, Miss Davidson, Bangour Hospital; Miss Thyne, West House; Miss Wise, Craig House. *Nursing Homes and Private Nurses*, Miss McGarth, 26 Chalmers Street; Miss Graham, 15 Alva Street; Miss Walker, 7 Rutland Square.

### Personalia.

The first Travelling Scholarship awarded by the People's League of Health in connection with the Sims Woodhead series of lectures on constructive educational health matters has been won by Mrs. M. D. Walters, a certified midwife and member of the Royal Sanitary Institute. Mrs. Walters will proceed to Belgium and make a report for the file of the League. The second course of lectures will begin in October; the fee is 10s., with 5s. examination fee, and full particulars can be had from the People's League of Health, 7 Hanover Square, W. 1.

### The Scottish Syllabus.

The Syllabus of Lectures and Demonstrations for Education and Training in General Nursing prepared by the General Nursing Council for Scoland is now ready for distribution. Applications for copies should be made to the Registrar, General Nursing Council for Scoland, 13 Melville Street, Edinburgh. The price is 1s. 2d. post free. The Syllabus will be required by all hospitals which desire to be approved as training-schools by the Council. A short Syllabus, intended for distribution by the hospital authorities to their probationers, has also been prepared by the Council, and is to be sold at a price of 2d. per copy in addition to postage.

### Dental Nurses in New Zealand.

The Education Department of the New Zealand Government has organised a scheme for training dental nurses for work amongst school children. The training is limited to specific parts only of dental work to enable them to carry out, under the supervision of registered dentists, the treatment of minor dental troubles in the younger school children. It is not intended that this training shall qualify dental nurses for registration as dentists, and registered dental surgeons will be employed for the higher class of dental work. Applicants for the dental nurse's training must be over twenty years of age, preference given to those between twenty-five and thirty-five. Accepted applicants are paid £90 per annum, with lodging allowance if away from home of £40 per annum. The full course of training is for two years. On qualifying the dental nurses are to receive a commencing salary of £220 per annum, rising by annual increments to £300, and will be required to sign an agreement for five years, the period of training being included. Most of the dental associations in the various centres throughout the Dominion are strongly objecting to and condemning the proposed scheme.

August 20 1921] [Price Twopence

### The Hospital

**RATES OF SUBSCRIPTION** (post free, payable in advance).
**Three months, 3/3; six months, 6/6; twelve months, 13/-. (Should the cost of printing and paper as well as increased postage, necessitate alteration of these rates, the period paid for will be reduced proportionately on unexpired subscriptions.) THE HOSPITAL "Index" to Volume LXIX., October 1920 to March 1921, is now ready, price 1/1 per copy, post free.** Address The Manager, THE HOSPITAL, 'The Hospital" Building, 28/29 Southampton Street, Strand, London, W.C. 2.

